# Pesticide Contamination of Honey-Bee-Collected Pollen in the Context of the Landscape Composition in Latvia

**DOI:** 10.3390/toxics12120862

**Published:** 2024-11-28

**Authors:** Niks Ozols, Valters Brusbārdis, Maksims Filipovičs, Jānis Gailis, Vitalijs Radenkovs, Betija Rubene, Viktorija Zagorska

**Affiliations:** 1 Institute of Plant Protection Research ‘Agrihorts’, Latvia University of Life Sciences and Technologies, 2 Paula Lejiņa Street, LV-3004 Jelgava, Latvia; makfil@lbtu.lv (M.F.); janis.gailis@llu.lv (J.G.); betija.rubene@lbtu.lv (B.R.); viktorija.zagorska@lbtu.lv (V.Z.); 2 Latvian Beekeepers Association, 22c Rīgas Street, LV-3004 Jelgava, Latvia; valters@strops.lv; 3 Institute of Horticulture (LatHort), 1 Graudu Street, LV-3701 Dobele, Latvia; vitalijs.radenkovs@lbtu.lv; 4 Division of Smart Technologies, Research Laboratory of Biotechnology, Latvia University of Life Sciences and Technologies, 22b Rīgas Street, LV-3004 Jelgava, Latvia

**Keywords:** Pollen Hazard Quotient, *Apis mellifera*, agricultural land, acetamiprid

## Abstract

The honey bee (*Apis mellifera*) is the most widely managed pollinator and is vital for crop fertilization. Recently, bee colonies have been suffering high mortality rates, exacerbated by factors such as land-use changes and the use of pesticides. Our work aimed to explore the residues of pesticides in honey-bee-collected pollen and how this contamination was affected by seasonality and the landscape composition. We selected six apiaries from different landscapes in Latvia, and pollen samples were collected during the flowering season (2023). We analyzed 39 samples and found 21 pesticide residues (mainly fungicides) with a frequency of 93 occurrences where the values were above the limit of quantification. The most frequently encountered substances were acetamiprid, boscalid, fluopyram, and prothioconazole. However, the highest concentrations were for dimoxystrobin (44 µg kg^−1^), acetamiprid (37 µg kg^−1^), azoxystrobin (27 µg kg^−1^), prothioconazole (25 µg kg^−1^), and boscalid (15 µg kg^−1^). We then calculated the Pollen Hazard Quotient (PHQ) for each pollen sample. No sample had a PHQ value above the critical value of 50. The highest contamination level was observed in the first half of the vegetation season (the end of May and the beginning of June), but later, it significantly decreased. We did not find any significant influence of landscape composition on pesticide pollution.

## 1. Introduction

Most recently, in Latvia and worldwide, the demand for crop pollination services has increased [1]; for example, the oilseed rape (*Brassica napus*) area in Latvia grew by 25% from the year 2011 to the year 2022. At the same time, many studies show that wild and managed bee species are decreasing [2,3,4]. A recent EU report showed that previously taken measures did not succeed in stopping the wild bee population decline [5].

As pollinators are essential for the reproduction of many wild plants and crops, it is crucial to find the main causes of the decline in pollinators. In the European report, the main causes mentioned are land-use changes for agriculture or urbanization, the use of pesticides, and invasion by alien species. In addition, genetically modified crops, habitat fragmentation, and introduced diseases and parasites also contribute to pollinator decline [6]. Honey bees (*Apis mellifera*) and bee products can be used as bioindicators of the presence of contaminants in the environment [7], and pesticides found in honey bee samples can be related to the potential contamination risk of wild bees as well.

Recently, an increase of 70% in pesticide sales compared with the year 2011 was reported for Latvia [8]. Most pesticides are used in agriculture, and the highest risk of contact with pesticides comes from contact with cultivated crops and places around agricultural fields. According to the sales data in Latvia, in 2022, the main groups of pesticides used were herbicides (60%), plant growth regulators (22%), fungicides (15%), insecticides, and acaricides (2%). Nevertheless, previous results show that fungicides and insecticides are the most frequently found pesticides in pollen samples [9]. These results can be explained by the fact that fungicides are mostly used just before or during the flowering of the crops. Insecticides are also often used during crop flowering. Although this is only allowed between 10 pm and 5 am, bees are still exposed to these pesticides. Insecticides and acaricides include compounds that pose major threats to arthropods, since they are designed to directly affect these, and for this reason, they have been extensively studied [6,10,11].

The amount of pesticides used changes during the season. Therefore, seasonality can play a strong role in increasing or reducing the level of contamination by pesticides in the pollen collected by honey bees [9]. Residues of agricultural pesticides in pollen can originate from the application of systemic compounds before the flowering period and from the contamination of water, soil, and other flowering strips beside the field, as well as from spray application to flowering plants [12].

Our objective in conducting this study was to analyze the pesticide contamination in pollen collected by honey bees and the potential risk to bee health in the context of landscape composition. Therefore, our tasks were (1) to identify pesticide residues in pollen; (2) to study the seasonal characteristics of the occurrence of these residues; (3) to analyze the potential risk to bee health caused by the presence of these residues in pollen; and (4) to find correlations between the landscape composition (proportion of agricultural land) and the pesticide risk to the bees. Before starting the research, we hypothesized that there is a relationship between the landscape composition around the apiary and the risk to bee health caused by pesticide residues: (1) as the proportion of agricultural land increases, pollen contamination with pesticides should also increase; (2) the greatest diversity and concentration of pesticide residues in pollen should be observed at the beginning of the growing season, from mid-May to mid-June, when various crops are in full bloom and when pesticides are most intensively used.

## 2. Materials and Methods

### 2.1. Study Area and Landscape Analysis

**Figure 1:**
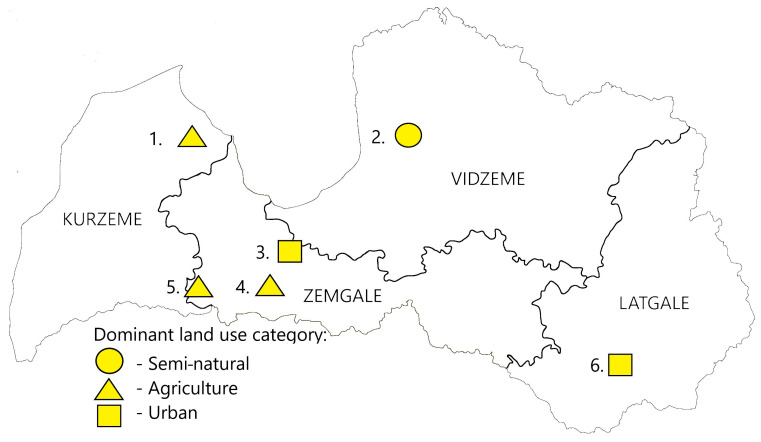
Pollen sampling sites (Semi-natural—forests, bushland, grasslands, waterbodies, and similar biotopes dominating the landscape; Agriculture—agricultural land dominating the landscape; Urban—built-up areas with infrastructure elements dominating the landscape; the numbers 1–6 coincide with the same numbers in Table 1).

We selected six commercial apiaries in different regions of Latvia (Figure 1) from which we collected pollen samples in 2023. Three apiaries were located in the Zemgale region—the most agricultural-intensive region in the country. The other three apiaries were located in Vidzeme, Kurzeme, and Latgale, respectively.

For each study site, we described the landscape within a three-kilometer radius (Table 1 and Table S1). Information about the agricultural land and specific crop areas was acquired from the Rural Support Service of Latvia. Data concerning the forest area were extracted from the State Forest Registry (georeferenced stand-level inventory database). The urban or built-up area cover was estimated using data from Dynamic World [13]; mean values from the year 2023 were extracted, and the area containing values in the category of “built-up area” were calculated. In this paper the “built-up area” category is called “urban”. Since we expected pesticide residues to mainly occur in agricultural land, we combined all forests, natural and semi-natural grasslands (originally belonging to agricultural land), waterbodies, and similar areas into a class called “semi-natural”. The area not belonging to either agricultural, urban, or forest areas was also assumed to be semi-natural. This resulted in three landscape classes: semi-natural, urban, and agriculture. The site was classified as a certain type if the proportion of that landscape class was more than 50%. In Jelgava town, the distribution between the semi-natural and urban land categories is nearly equal. This is attributed to Jelgava’s extensive green spaces, which includes parks, squares, and trees. Despite having only 44% of the area classified as “built-up,” the town is still categorized as urban due to the apiary location.

In Table 1 and Figure 1, information about the distribution of areas according to the classification “agriculture”, “semi-natural” and “urban” can be seen. The cover of semi-natural areas ranged from 17 to 72% (mean = 48%), the cover of agricultural areas ranged from 7 to 80% (mean = 42%), and the cover of urban areas ranged from 0 to 44% (mean = 10%). Lists of the biotopes surrounding the study sites are given in Table S1.

### 2.2. Pollen Sampling and Palynological Analysis

From May to August, pollen was collected from the apiaries using pollen collectors every second day or at longer intervals, depending on the weather conditions. Pollen damaged on rainy days was not collected. The damaged content was disposed of, and the pollen collectors were cleaned with 70% ethanol solution in preparation for the next pollen collection. In each apiary, pollen was taken from two marked hives, which were then mixed together as a one-day sample (100 g). Each sample was labelled with the name of the apiary and the date of collection. After collecting samples from apiaries, a thermal cold-box with cooling elements were used for temporary storage and transportation to the lab; samples were then stored in a freezer at −18 °C. Then, samples were processed for palynological and pesticide residue analysis. The sampling season was divided into seven two-week periods: 15–28 May (period 1); 29 May–11 June (period 2); 12– 25 June (period 3); 26 June–9 July (period 4); 10–23 July (period 5); 24 July–6 August (period 6); and 7–20 August (period 7). All one-day samples collected from each apiary in each period were combined into one two-week sample. This resulted in 39 pollen samples being analyzed—7 from each site, except for the Platone parish apiary, where the bees stopped collecting pollen at the end of June, so only 4 two-week samples were analyzed from this site.

Determination of the botanical composition of the pollen in the samples was conducted at the Institute of Plant Protection Research “Agrihorts” laboratory. A modified methodology of light microscopy was used [14,15]. Pollen identification and counting were performed on images obtained by a light microscope with 400× magnification. On each sample, three replicates were performed, with 8–10 images per replicate and 10–40 pollen grains in each picture, resulting in a total of >500 identified pollen grains.

### 2.3. Chemical Analysis of Pollen Samples

Commercial standards, i.e., acetamiprid, phosphonic acid (AMPA), azoxystrobin, bixafen, boscalid, cyprodinil, deltamethrin, difenoconazole, diflufenican, epoxiconazole, florasulam, fluopyram, fluroxypyr, fluxapyroxad, iodosulfuron-methyl-sodium, MCPA, metazachlor, metconazole, metrafenone, pendimethalin, prosulfocarb, pyraclostrobin, spiroxamine, tebuconazole, and trinexapac-ethyl were purchased from Sigma-Aldrich Chemie Ltd., (Sigma, St. Louis, MO, USA). Methanol (MeOH), acetonitrile (MeCN), and formic acid (HCOOH) (puriss p.a., ≥99.9%) of liquid chromatography–mass spectrometry (LC-MS) grade were purchased from Merck KGaA (Darmstadt, Germany). The “SupelTM-Swift HLB” (57492-U) solid-phase purification column packed with a hydrophilic modified, styrene-based sorbent (50–70 µm, 80–200 Å, 60 mg 3.0 mL) was obtained from Supelco^®^ (Bellefonte, PA, USA). Ultrapure water (UPW) was produced using the reverse-osmosis PureLab Flex Elga water purification system (Veolia Water Technologies, Paris, France).

A modified method known as the “quick method for the analysis of highly polar pesticides in food (QuPPe-PO)” was employed to extract pesticides from the pollen samples [16]. In short, the wet pollen samples were frozen using liquid nitrogen and subsequently crushed uniformly to form a homogeneous fraction using a granite pestle and stamp. Then, 2 g (to an accuracy of 0.01 g) of the crushed sample was transferred into a 50 mL centrifuge tube (Sarstedt AG & Co. KG, Nümbrecht, Germany), and 5.0 mL of acidified UPW H_2_O:HCOOH (pH 2.2 ± 0.2) (ratio H_2_O:HCOOH, 99:1 *v*/*v*) was added. The mixture was intensively vortex-mixed for 2 min with the “ZX3” vortex mixer (Velp^®^ Scientifica, Usmate Velate, Italy) followed by ultrasonic treatment at 50 kHz with an output wattage of 360 W for 15 min at 20 ± 1 °C using an “Ultrasons” ultrasonic bath (J.P. Selecta^®^, Barcelona, Spain). After completion of the treatment, 5.0 mL of acidified methanol MeOH:HCOOH (pH 2.2 ± 0.2) (ratio MeOH:HCOOH, 99:1 *v*/*v*) was introduced into the tube, and the contents of the tube was intensively vortex-mixed for 2 min and subjected to additional ultrasonic treatment for 15 min at 20 ± 1 °C. Afterwards, the obtained mixture was subjected to freezing for 30.0 min at −18 ± 1 °C, and the pre-cooled mixture was centrifuged at 9280× *g* for 10 min at −10 ± 1 °C in a “Hermle Z 36 HK” centrifuge (Hermle Labortechnik, GmbH, Wehingen, Germany). The obtained supernatant was decanted and filtrated through a 0.45 µm polytetrafluoroethylene (PTFE) hydrophilic membrane filter (Macherey-Nagel GmbH & Co., KG, Dueren, Germany). Prepared filtrates were kept at a temperature of −18 ± 1 °C until further analysis and used within a maximum of three days.

**Figure 2:**
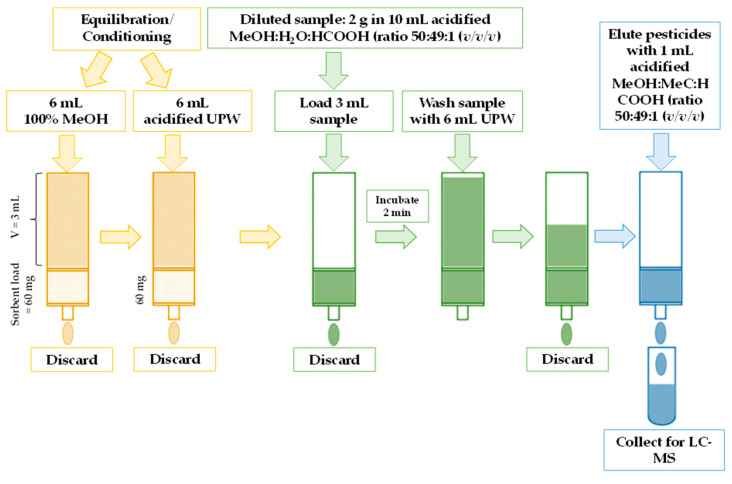
A schematic representation of the simplified solid-phase extraction clean-up procedure for pesticides in the pollen samples using by a styrene-based sorbent approach.

Purification of pesticides for further LC-ESI-TQ-MS/MS analysis was approached by using a “SupelTM-Swift HLB” (57492-U) (Supelco, Bellefonte, PA, USA) solid-phase extraction (SPE) column packed with a hydrophilic modified styrene-based sorbent (50–70 µm, 80–200 Å, 60 mg, 3.0 mL) (Figure 2). The selection of sorbent was based on a preliminary study conducted with the utilization of four commercially available SPE columns, i.e., “Strata-X” (Phenomenex, Torrance, CA, USA), “SupelTM-Select HLB” and “SupelTM-Swift HLB” (Supelco, Bellefonte, PA, USA), and Chromabond^®^ HR-X (Macherey-Nagel GmbH & Co., KG, Dueren, Germany). The highest recovery of a particular pesticide was reached when “SupelTM-Swift HLB” was applied (Supplementary Materials Figure S1), due to the sorbent possessing both hydrophilic and lipophilic functional groups. A steady flow (1.0 ± 0.2 mL min^−1^) during analyte desorption was provided by a “Chromabond^®^ SPE” (Düren, Germany) SPE vacuum manifold with an adjusted pressure of 3.38 × 10^−3^ Pa. The conditioning/equilibration of the SPE column was performed using 2 bed volumes (3.0 mL) of pure MeOH followed by 2 bed volumes of acidified UPW (1.0% HCOOH, *v*/*v*). The loaded extract (3.0 mL) was washed with 2 bed volumes of UPW.

The flow-through fractions were collected for qualitative (screening) and quantitative chromatographic analysis for the presence of residual pesticides. One mL of the acidified MeOH:MeCN:HCOOH (ratio 50:49:1 (*v*/*v*/*v*)) solution was used as an eluate to elute pesticides from the polymer. The resulting fractions were collected and analyzed with an LC-ESI-TQ-MS/MS system.

**Figure 3:**
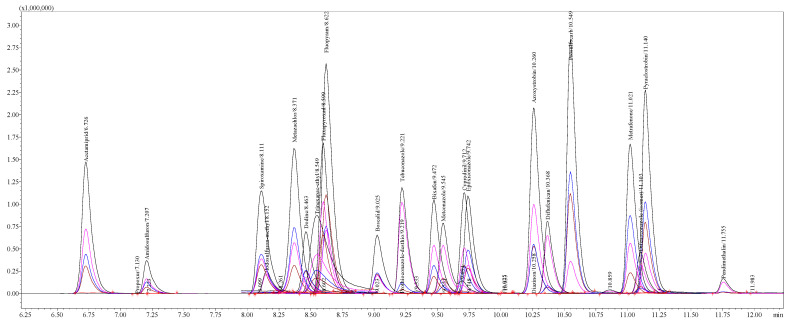
Extracted-ion chromatogram (EIC) in multiple-reaction monitoring (MRM) mode representing the profile of major pesticide standards at the concentration of 1 μg mL^−1^.

The analysis was approached by a Shimadzu series “Nexera UC” supercritical fluid extraction–supercritical fluid chromatography–mass spectrometry (SFE-SFC-MS) system (Shimadzu Corporation, Tokyo, Japan) coupled to a Shimadzu triple quadrupole (TQ) mass-selective detector (TQ-MS-8050) (Tokyo, Japan) with an electrospray ionization interface (ESI). Chromatographic separation of residual pesticides was carried out using the “Restek Raptor™ Biphenyl” column (2.1 μm, 100 × 2.7 mm; Restek, Bellefonte, PA, USA) operating at 35 °C and a flow rate of 0.4 mL min^−1^. The mobile phases used were acidified UPW (0.002% HCOOH, *v*/*v*) supplemented with 2 mM ammonium formate (A) and acidified MeOH (0.002% HCOOH, *v*/*v*) with 2 mM ammonium formate (B). Compounds were separated using the stepwise gradient elution program: elution started with 3% B to obtain 10% B at 1.0 min, 55% B at 1.0–3.0 min, 100% B at 10.5–12.0 min, and 3% B at 12.01 min with a subsequent 3 min wash and equilibration. Furthermore, MeOH injections were included as a blank run after each sample to avoid a carry-over effect. Data were acquired using “LabSolutions Insight” LC-MS software version 3.7 SP3 (workstation), which was also used for instrument control and processing. The operating conditions were as follows: detector voltage, 1.8 kV; conversion dynode voltage, 10.0 kV; interface voltage, 3.0 kV; interface temperature, 350 °C; desolvation line temperature, 150 °C; heat-block temperature, 300 °C; nebulizing gas, argon (Ar, purity 99.9%), at a flow rate of 3.0 L min^−1^; heating gas, carbon dioxide (CO_2_, purity 99.0%), at a flow rate of 10.0 L min^−1^; and drying gas, nitrogen (N_2_, separated from air using a nitrogen generator system from “Peak Scientific Instruments Ltd.” (Inchinnan, Scotland, UK); purity 99.0%) at a flow rate of 10.0 L min^−1^. Qualitative analysis of the pesticides was performed in the programmed and optimized multiple-reaction monitoring (MRM) mode following the method developed by Shimadzu Corporation (Tokyo, Japan) for the analysis of 646 pesticides using Multi-Residual Pesticides Ver. 3 [17]. Quantification was performed using reference standards, building calibration curves for each pesticide compound individually (Supplementary Materials; Figure S2). A representative chromatographic separation of the major pesticides used for building the calibration curves is shown in Figure 3.

The validation experiment was carried out according to the EU Reference Laboratory for Pesticides No. SANTE/12682/2019 guidelines [18] to estimate the linearity, matrix effect, limit of detection (LOD) and quantification (LOQ), sensitivity, accuracy, and precision. Pollen from organic beekeeping was used as a blank to prepare the pollen matrix-matched calibration. Linearity was evaluated both in pure solvent and the pollen matrix, using seven-point matrix-matched calibration levels (R^2^ > 0.99) within the range of 0.6–160 ng mL^−1^ (Supplementary Materials; Figure S2). The matrix effect of the pollen extracts was evaluated by comparing the slopes of the calibration curves in pure solvent MeOH:MeC:HCOOH (ratio 50:49:1 (*v/v/v*)) with those in the respective matrix. Recovery (%) and precision (RSD%) were assessed by fortifying blank pollen samples at three concentration levels of the test analytes for three replicates (Supplementary Materials; Figure S1). Recoveries between 70–120% with an RSD < 20% were considered satisfactory [18]. Method sensitivity was evaluated by calculating the limit of quantification (LOQ) as the minimum amount of analyte detected with a signal-to-noise ratio of 10 and the limit of detection (LOD) as the minimum amount of analyte detected with a signal-to-noise ratio of 3 (Supplementary Materials; Table S2). Concentrations were determined using a least-squares linear regression analysis of the peak-area ratio versus the concentration ratio of a particular reference standard.

### 2.4. PHQ Values as a Response Variable

We used Pollen Hazard Quotient (PHQ) values [19] as a measure of the potential pollen toxicity for honey bees. The PHQ is calculated as the ratio between the compound concentration in ppb (μg kg^−1^) and the oral or contact LD50 for honey bees [12]. Based on a daily consumption of up to 9.5 mg of bee bread by a nurse bee [19], a PHQ of ≥50 is considered “relevant” for bee health, according to [20].

To assess the impact of agricultural landscape cover on the PHQ, we conducted a simple linear regression with agricultural land cover (%) within a 3 km radius around the apiaries as a factor and PHQ values as the response variable (R version 4.4.0.; R Core Team Vienna, Austria, 2024). PHQ values from periods three to seven were near zero, and so only data from the first and second sample period were used in the model (*n* = 12). Over-dispersed data and low power in the model meant that we used a largely descriptive approach in our interpretation of the results.

In addition, we divided all the crops in the landscape depending on the general use of pesticides reported in Latvia [21,22]. The groups used were “none” (no pesticides used), “low” (<0.5 kg a.s. ha^−1^; a.s.—active substance), “moderate” (0.5–1.0 kg a.s. ha^−1^), and “high” (>1.0 kg a.s. ha^−1^) (Table S3). The groups were used to analyze the effect of both total agricultural land and nectar crop area on the PHQ. In our case, the “nectar plants” are all the cultivated plants visited by the bees for collecting nectar and/or pollen. Since bees are known to be the main pollinators of oilseed rape (*Brassica napus*) [23], apple orchards (*Malus domestica*) [24], and field bean (*Vicia faba*) [25], we also analyzed whether the area of these specific crops helped to explain the differences in PHQ values.

## 3. Results and Discussion

### 3.1. Active Substances Detected and Quantified in the Pollen Samples

In 2023, a total of 21 active substances containing pesticide residues were detected at levels above the limit of detection (LOD), with 19 of these substances being quantified above the limit of quantification (LOQ). The most commonly found pesticides in the pollen samples were fungicides, with 12 active substances detected. Among them, boscalid, fluopyram, prothioconazole, azoxystrobin, and tebuconazole were observed most frequently. Additionally, four herbicides were found, but their frequencies were relatively low. Two insecticides were also detected. One of them, acetamiprid, was the most frequently found active substance among all the pesticides detected in the analyzed samples (see Table 2). Our results are similar to observations elsewhere in Europe. In Switzerland, in 2020, the most prevalent pesticides were fungicides (particularly azoxystrobin and boscalid), followed by herbicides (such as prosulfocarb and terbuthylazine) and insecticides (especially thiacloprid) [26]. Similarly, in Germany in 2019, the highest concentrations detected were of the fungicides azoxystrobin, boscalid, prosulfocarb, and trifloxystrobin, with maximum concentrations of 482, 339, 106, and 136 μg kg^−1^, respectively [20].

The most prevalent substance (>LOD) found in our pollen samples was the neonicotinoid insecticide acetamiprid (max. 37 μg kg^−1^). In comparison with other literature sources, acetamiprid has been detected at various concentrations in different regions. In Switzerland, Schaad et al. [26] reported a concentration of 16 μg kg^−1^. In Portugal, Capela et al. [27] found acetamiprid levels in pollen samples at concentrations ranging from 15 to 592 μg kg^−1^ one day after spraying. In South Germany, Friedle et al. [12] reported concentrations ranging from 0.02 μg kg^−1^ to 30.75 μg kg^−1^. In China, Tong et al. [28] detected concentrations between 5.2 μg kg^−1^ and 63.6 μg kg^−1^. This means that the concentrations of acetamiprid detected in our study fall within the range of previously published data and do not exceed those boundaries; however, comparing data from different sources is challenging. Each study involved varying methodologies, including differences in the pollen sample collection times, frequencies, and periods. Additionally, whether the samples were analyzed as dry or wet and whether they were collected based on a timeline or color similarities varied depending on the specific objectives of each research study.

Dimoxystrobin was the pesticide with the highest concentration observed in the pollen samples we analyzed (max 44 μg kg^−1^). In comparison, Hungarian market-shelf samples contained dimoxystrobin at a level of 276.38 μg kg^−1^ per dry matter, which is approximately six times higher [29]. In South Germany, the maximum concentration recorded was 576.2 μg kg^−1^ [20], which is approximately 13 times higher than our maximal concentrations.

In our study, the maximum concentration of azoxystrobin detected in the pollen samples was 26 μg kg^−1^. Comparatively, in other studies, concentrations of this active substance have varied widely. For example, in Luxemburg, azoxystrobin concentrations varied from 0.29 till 201.81 μg kg^−1^ [30]. In Germany, it accounted for a maximum value of 560.5 μg kg^−1^ [20]. These findings highlight the variability in the presence of azoxystrobin in different environments and the relatively low levels found in our samples.

The maximum concentration of prothioconazole in our pollen samples was 25 μg kg^−1^. This widely used fungicide has been detected in pollen samples in various studies, raising concerns about its impact on bee health and the broader environment. For example, a study conducted in Poland found that prothioconazole residues in bee pollen reached concentrations as high as 356.7 μg kg^−1^ [31], but in Switzerland, it was 5–1000 μg kg^−1^ [26], which exceeds the EU’s maximum residue limit for this substance. This highlights the significant exposure risks for bees in regions where prothioconazole is extensively used. Exposure to this fungicide can cause changes in the expression of crucial health-related genes in honey bees, particularly when it is combined with other fungicides such as bixafen and trifloxystrobin. These changes in genetic expression can affect bees’ detoxification processes and immune responses, especially under conditions of nutritional stress [32]. This underscores the complex interplay between pesticide exposure and bee health, suggesting that while prothioconazole is effective for agricultural disease management, its use in environments frequented by pollinators requires careful monitoring and potentially stricter regulation to prevent adverse ecological effects [33].

In some pollen samples, two pesticides—the insecticide thiacloprid and the fungicide propiconazole—were found. Both of them have been banned for use in Latvia since 2020. It means that despite this restriction, thiacloprid was illegally applied in 2023. The decomposition period of this chemical is comparably short—only eight days (DT50 field) [34]—therefore, it is unlikely that we observed historical contamination of this active substance. Propiconazole is not allowed in agriculture. However, this fungicide is still encountered in wood protection products; therefore, exposure could come through some wooden parts. In 2024, the European Commission decided to renew the approval of propiconazole despite its potential risks because the fungicide is crucial for temporary treatment against wood-discoloring fungi and the treatment of structural wood and joinery.

### 3.2. Pollen Contamination According to Seasonality and Landscape Composition

**Figure 4:**
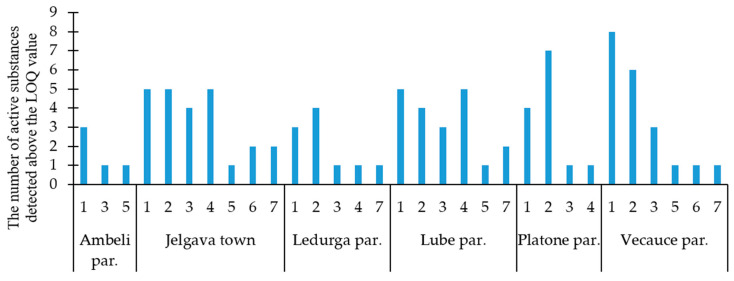
The number of detected active substances in the pollen collected during the sampling periods in 2023. (If a period is not specified for a site, it means that no pesticides were detected in the pollen during that period).

The number of pesticides identified per pollen sample ranged from zero to eight (see Figure 4). The highest diversity of pesticides was observed at the beginning of the sampling season—during the first and the second period. For example, in the apiaries located in the Vecauce parish, eight different pesticides were detected during the first period, which was the highest number found in a single sample at one location throughout the entire research season. In comparison, a smaller pesticide load was found in the pollen from the apiary of the Ambeli parish. At this location, three different active substances were detected in the first period, but no pesticide contamination was observed in the second period. In addition, in the following research season, pesticide presence in pollen was mostly not detected at this location.

**Figure 5:**
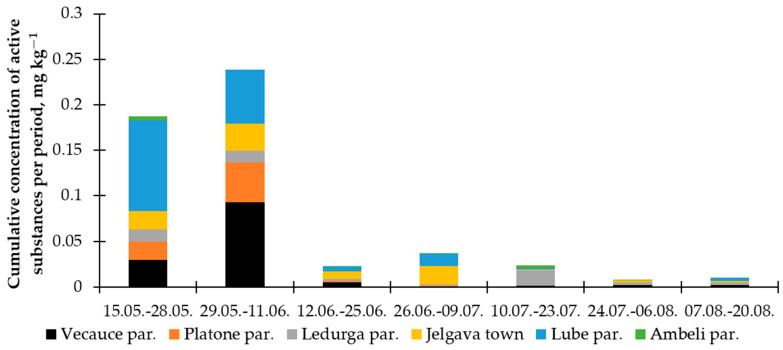
The cumulative concentration of active substances in the pollen per period, divided by location.

The highest cumulative pesticide concentration was found during the first and the second sampling periods in the Vecauce parish and the Lube parish (see Figure 5). For both locations, pollen of crucifers dominated in the analyzed samples (see Table 3 and Table S4). It is likely that these were mostly winter oilseed rape because the flowering period of this plant in Latvia coincides with the first two periods of our observations. The oilseed rape is very attractive to honey bees, and it is visited more often despite the presence of other flowering crops or wild plants in the landscape [35].

In the Platone parish, the greatest expanse of winter oilseed rape, covering 305 ha within a 3 km radius, was observed. However, while this area exhibited the highest quantity of Brassicaceae pollen concentration among the six sampling locations (Table 3), it did not record the highest cumulative pesticide concentration (Figure 4). This apparent contradiction could be ascribed to two potential explanations. First, the concentration levels of pesticides in the pollen samples may be influenced by farmers’ spraying practices, including the timing and frequency of the pesticide application. Second, it is plausible that pollen samples of the Brassicaceae family do not exclusively represent oilseed rape; therefore, a pollen sample analysis at the species level is needed.

In other apiaries in the study, relatively low contamination of pollen with pesticide residues was observed in all periods, as well as in the apiaries of the Vecauce and Lube parish from the third period onwards. This is most likely due to the reduction in the need for pesticide use in Latvia from mid-June onwards. As well as cultivated arable crops and orchards, wildlife in grasslands, roadsides, and other such places where pesticides are not sprayed is intensely blooming during this time. For example, in period 4, 53.6% from the Asteraceae family (see Table S4) dominated the pollen collected by bees in the Lube parish apiary, from which only some medicinal and ornamental plant species, grown in small areas, were cultivated in Latvia. It is therefore very likely that the bees mostly visited pesticide-free biotopes.

**Figure 6:**
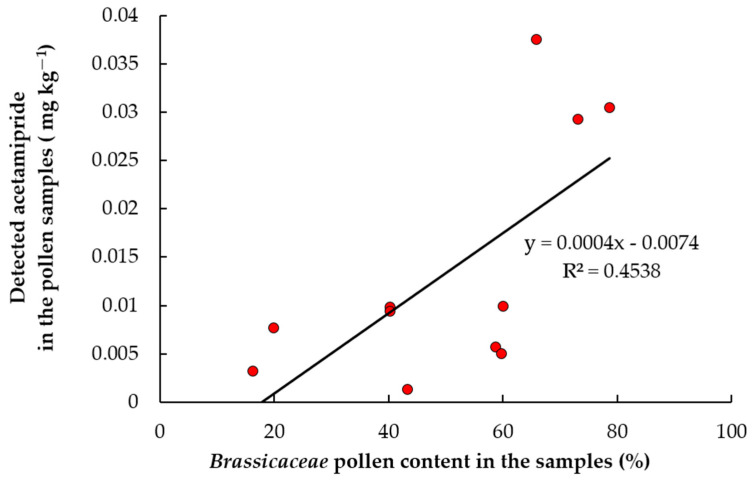
Relationship between acetamiprid contamination and the proportion of crucifers (*Brassicaceae*) pollen in the studied apiaries.

Since acetamiprid was the most frequently found pesticide in our samples during the first two observation periods, we tried to find relationships between its presence in pollen and landscape composition. We first looked at the correlation between the concentration of this insecticide in pollen and the proportion of agricultural land within a radius of three kilometers around the apiaries. However, we did not find a statistically significant relationship either in the first (r = 0.193, *p* = 0.715) or second (r = 0.309, *p* = 0.551) observation period. We then looked for a relationship between acetamiprid concentration and the proportion of crucifers’ pollen in the samples, and this turned out to be significant (r = 0.67, *p* = 0.05) (Figure 6). This relationship can be explained by several circumstances. This pesticide is the most popular systemic insecticide left in the Latvian registration since 2022. It is approved for use in the control of a variety of pests among most crops grown in the country, including the pollen beetle (*Brassicogethes aeneus*) and *Ceutorhynchus* weevils, which are important oilseed rape pests that are mostly controlled in the first part of the growing season. We discussed earlier that the crucifers’ pollen in our samples might have mostly come from oilseed rape fields. Therefore, we can conclude that the concentration of acetamiprid in the pollen collected by honey bees does not depend on the total proportion of agricultural land in the landscape but is determined by the availability of flowering oilseed rape fields.

### 3.3. Potential Hazard to Honey Bees (PHQ Values)

**Figure 7:**
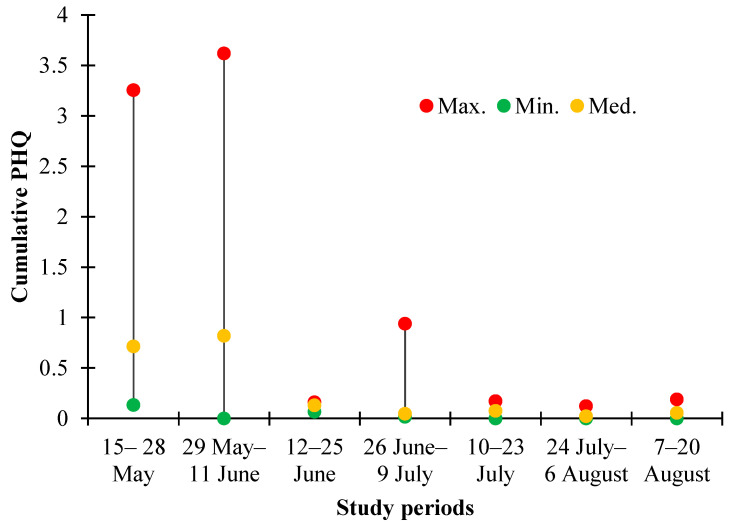
Variation of the cumulative Pollen Hazard Quotient (PHQ) in the studied honey bee apiaries in 2023.

None of the analyzed pollen samples had a cumulative PHQ value that exceeded the critical acute toxicity threshold. The highest concentrations of pesticides in pollen were observed in the first two periods of the study—the second half of May and the beginning of June. During these periods, the highest PHQ values were also obtained (Figure 7). Although different fungicides were found in higher concentrations and numbers in the pollen, insecticides had a greater weight in the cumulative value of the PHQ (Table S5). For example, in the first and second periods of the study, insecticides accounted for 45–97% of PHQ in different apiaries; it should be added that it was usually only one active substance—acetamiprid. Herbicides had an insignificant effect on PHQ formation, as residues of these pesticides were observed infrequently and at low concentrations in the pollen. The essential role of insecticides in the formation of the PHQ can be explained by a simple circumstance. These active substances are synthesized to kill insects. Their LD50 values for bees are significantly lower compared with those for fungicides and herbicides [36].

The highest cumulative values of the PHQ in the first periods of the study were found in two agricultural regions. In the first period, the highest PHQ (3.256) was found in the Lube parish, while the Vecauce parish had the second highest PHQ value (1.035). In the second period, the highest PHQ was found in the Vecauce parish (3.621), while the Lube parish had the second highest PHQ value (2.358). In both cases, these PHQ values were significantly higher than the median values. Elsewhere in the first two study periods, the PHQ values were equal to or less than the median value of less than 1.000. Beginning with the third study period, the pesticide concentrations in pollen were so low that it was not possible to identify any region with a significantly higher PHQ compared with other places. However, the fact that the apiary is located in a region where the landscape is dominated by agricultural land does not automatically mean that the pollen will be more contaminated with pesticides. For example, in the apiary of Platone parish, the cumulative value of the PHQ was equal to or less than the respective median value in all the study periods.

Although the PHQ values in our study were below the threshold level, it cannot be ruled out that honey bees and wild pollinators in Latvia are exposed to a chronic sublethal pesticide risk. Moreover, in cases where each pesticide on its own does not, to our knowledge, have a significant negative effect on bees, their combinations may nevertheless have a negative impact. Acetamiprid, the most common insecticide in our analyzed samples, belongs to the neonicotinoid group. In many parts of the world, after the active use of neonicotinoids in agriculture began, beekeepers observed atypical bee behavior; including bees having difficulty navigating and finding their hive. The winter mortality rates of bee colonies also increased. It is known that neonicotinoids in sublethal doses negatively affect honey bee behavior and immunity to pathogens. In cases of chronic sublethal contamination, honey bees have been observed to have a reduced learning capacity and other undesirable effects [37]. This can be explained by the fact that neonicotinoids, including acetamiprid, significantly affect the physiological activity of the central nervous system of honey bees at sublethal doses [38]. Various studies have also observed the sublethal effects of acetamiprid on wild bees. For example, acetamiprid alters the foraging behavior of buff-tailed bumblebee (*Bombus terrestris*) workers. They have been observed to have delayed foraging [39] or to be completely unable to find food resources [40]. At sublethal doses, two neonicotinoid insecticides, thiamethoxam and clothianidin, reduced the reproductive performance of the red mason bee (*Osmia bicornis*) by 50%, with the majority of offspring developing as males [41]. In turn, a sublethal dose of acetamiprid in combination with the fungicide tebuconazole significantly affected the behavior of the mason bee *Osmia cornuta*. Bees had difficulty finding their nesting cavities, which reduced the provisioning rate and reproductive success. When bees were exposed to any of the pesticides alone, no such negative effects were observed [42].

### 3.4. The Impact of Landscape Composition on the PHQ

**Figure 8:**
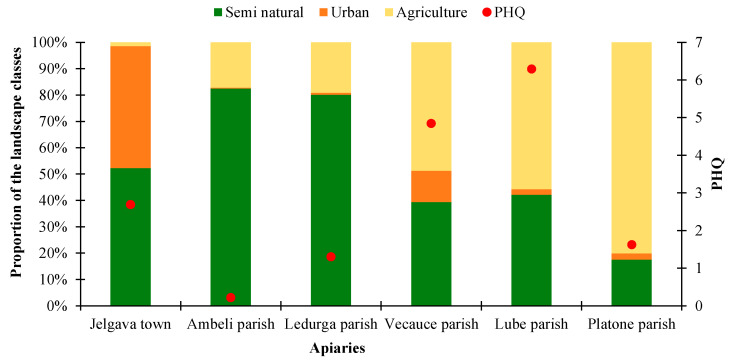
Cumulative PHQ values and the landscape composition within a 3 km radius for each apiary in 2023.

We expected an increase in the total PHQ in areas with a higher proportion of agricultural land. As can be seen from Figure 8, this assumption holds in four of the studied regions, except in cases with the highest and the lowest crop percentages. Although close to the significance level, we found no significant association between the percentage of agricultural lands and the PHQ (β = 0.06; *p* = 0.07).

**Figure 9:**
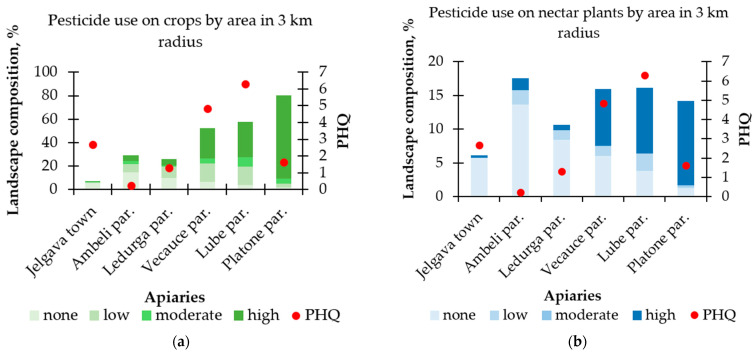
Cumulative PHQ and landscape composition within a 3 km radius around the apiaries in 2023. (**a**) All agricultural land; (**b**) Land of cultivated nectar plants. In both cases, the proportion of land according to the amount of pesticide used is shown.

When the agricultural area was divided into groups by pesticide use, no clear explanation could be found for Jelgava town and Platone parish as to why their PHQ values were far from the expected results. The area around Jelgava town had the lowest proportion of agricultural land, but the PHQ found in this apiary was one of the highest. The opposite was the case in the Platone parish, where the area around the apiary had the largest proportion of agricultural land, most of which were used for crops, in the cultivation of which a lot of pesticides were used, judging by official statistics. However, this site was found to have one of the lowest PHQs (Figure 9a). Pesticide use on nectar plants specifically also did not show anything that could aid in resolving the ambiguous results of Jelgava town and the Platone parish (Figure 9b).

**Figure 10:**
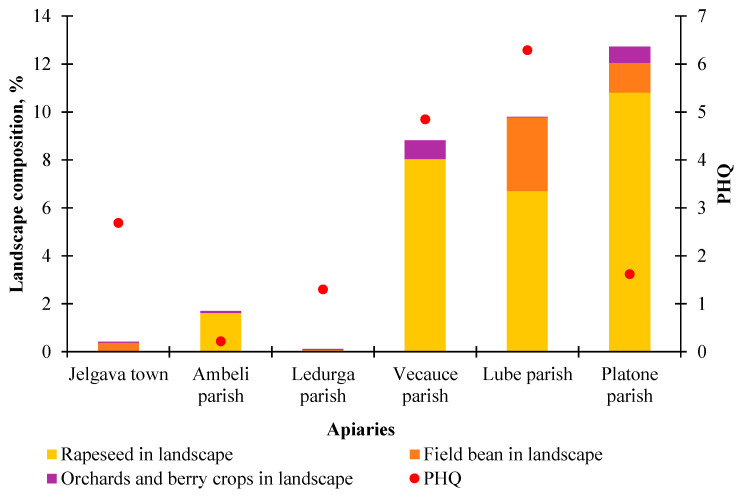
Specific crop-type areas (oilseed rape, field bean, and orchards/berries) within a 3 km radius around the apiaries and their cumulative PHQ values in 2023.

Since neither the total proportion of agricultural land nor its division by the amount of pesticide use allowed us to find an explanation for the PHQ values, we paid attention to specific crops: the proportion of winter rape, field beans, and orchards/berry crops in the landscape composition around the studied apiaries. These crops flower alternately from the second half of May to mid-June, when our pollen samples had the highest contamination with pesticide residues. However, this approach also did not show a meaningful relationship between the proportion of any crop and the PHQ. For example, the Vecauce and Lube parishes had a large proportion of these crop areas in the landscape, and their PHQ was the highest. However, the proportion of these plants was the highest in the Platone parish, where the PHQ was comparably low. In the apiary of Jelgava town, a moderately high PHQ was found, but winter rapeseed, field beans, and orchards/berry crops were almost absent in the landscape (Figure 10).

Our results indicate that agricultural land area alone is not sufficient to predict the presence of pesticide residues in honey bee pollen. Information about the amount of pesticides used in specific crop types also did not explain the differences between the PHQ values found in the studied sites. Even though the PHQ values and crop area seem to be connected in four of the study sites, two sites exhibited strong differences to the expected results, so our hypothesis was rejected. This is in opposition to other studies that found a clear relationship between the PHQ and agricultural land area in the landscape [9].

We propose two possible explanations for this finding. The first one is pesticide-use differences in the same type of crops. Pest and pathogen activity may vary depending on local environmental conditions. Therefore, when growing the same crops, pesticide application can vary significantly between different locations within the same year. The other factor likely to influence PHQ values is the honey bee foraging behavior. It is still not clear how bees decide on their foraging resources—some authors note that bees prefer to forage in agricultural lands [43], while some report increased foraging in urban landscapes [12]. Most authors also add that their results should be interpreted in the specific context studied; honey bee foraging choices are evidently dependent on the different conditions and resource availability at a specific site, thus each case should be looked at individually. Foraging activity is also a factor that needs to be considered, as the foraging intensity right after pesticide spraying could have an impact on how much of the pesticides the bees are exposed to. Friedle et al. [12] have reported that pesticide residues can differ greatly between adjacent days. Therefore, many factors could have influenced the PHQ, and it is hard to pinpoint any one of these, as the foraging behavior of the bees was not looked at closely in this study. It is also likely that with more samples, we would have achieved a higher statistical power in the model and a more precise view of the associations between landscape composition and the PHQ.

## 4. Conclusions

Residues of active substances of 21 pesticides were found in the pollen collected by honey bees in Latvia. Most of them were different fungicides, but the insecticide acetamiprid was most often detected in the analyzed samples. The highest cumulative concentrations of pesticide residues were found at the beginning of the vegetation season—from mid-May to mid-June—just as we hypothesized before the study was conducted. A strong positive correlation was observed between the crucifer (*Brassicaceae*) pollen proportion and the acetamiprid concentration in the samples; this leads to the conclusion that these pollen were mostly collected in rapeseed fields.

No significant acute risk to the health of honey bees was found—PHQ values in all the samples were below the threshold. Contrary to our hypothesis, we did not find a statistically significant relationship between the PHQ and the landscape composition—an increase in the proportion of agricultural land in the landscape did not correlate with a higher concentration of pesticides in the pollen and a higher PHQ. Also, a small proportion of agricultural land in the landscape does not guarantee that honey bees will be able to not come into contact with pesticides when collecting pollen and nectar.

## Figures and Tables

**Table 1:** List of the six sampling locations (apiaries), with information on the percentage of semi-natural, agricultural, and urban land within a 3 km radius around the apiaries.

No.	Sampling Location (Apiary)	Semi-Natural, %	Agriculture, %	Urban, %
1	Lube parish, Kurzeme	40	58	2
2	Ledurga parish, Vidzeme	72	27	1
3	Jelgava town, Zemgale	49	7	44
4	Platone parish, Zemgale	17	80	2
5	Vecauce parish, Zemgale	37	52	11
6	Ambeli parish, Latgale	70	29	0

**Table 2:** Active substances detected in the pollen samples in 2023.

Active Substance Name	Pesticide Class *	Max Concentration, μg kg^−1^	Min Concentration, μg kg^−1^	LOD, μg kg^−1^	LOQ, μg kg^−1^	Frequency (Number of Samples) (>LOD)
Acetamiprid	I	37	1	0.22	0.68	28
Azoxystrobin	F	27	1	0.07	0.23	7
Bentazone	H	10	10	0.16	0.48	1
Bixafen	F	0.1	0.10	0.24	0.72	1
Boscalid	F	15	1	0.21	0.64	10
Cyprodinil	F	0.3	0.3	0.08	0.26	1
Difenoconazole	F	3	2	0.26	0.78	2
Dimoxystrobin	F	44	1	0.07	0.22	5
Epoxiconazole	F	0.04	0.04	0.14	0.43	1
Florasulam	H	1	1	−	−	1
Fluopyram	F	15	0.4	0.04	0.13	8
Metazachlor	H	1	1	0.05	0.17	1
Metconazole	F/R	1	1	0.06	0.18	2
Pendimethalin	H	0.3	0.3	0.08	0.26	2
Picloram	H	1	1	0.08	0.26	1
Propiconazole	F	2	2	0.14	0.43	1
Prothioconazole	F	25	1	0.09	0.29	8
Pyraclostrobin	F	3	1	0.17	0.52	4
Spiroxamine	F	1	1	0.16	0.48	2
Tebuconazole	F	2	0.5	0.09	0.29	7
Thiacloprid	I	2	2	0.22	0.68	2

* F—fungicide; I—insecticide; H—herbicide; R—growth retardant.

**Table 3:** Content of Brassicaceae pollen in the samples vs. the area of winter oilseed rape around the apiaries in 2023.

	Ledurga Par.	Ambeli Par.	Lube Par.	Platone Par.	Jelgava Town	Vecauce Par.
Winter oilseed rape within a 3 km radius, ha	2	12	189	305	0	227
Brassicaceae pollen content (%) in the pollen samples (1st/2nd period *)	59/60	43/57	66/73	16/60	20/40	40/79

* 1st period—15–28 May; 2nd period—29 May–11 June.

## Data Availability

The data used to prepare the article are included in the tables, figures, and Supplementary Materials. For more detailed information, do not hesitate to contact the corresponding author.

## Supplementary Materials

The following supporting information can be downloaded at: https://www.mdpi.com/article/10.3390/toxics12120862/s1, Figure S1: Recovery of pesticides; Figure S2: Recovery curves; Table S1: Biotopes surrounding the study sites; Table S2: Performance of the analytical method for a particular pesticide in the pollen; Table S3: Cultivated plants within a radius of 3 km around the studied apiaries; Table S4: Pollen composition analysis by sampling periods in 2023; Table S5: Calculated PHQ in pollen samples from 2023.
