# Supplementary material for: Pesticide Contamination of Honey-Bee-Collected Pollen in the Context of the Landscape Composition in Latvia
Source: Toxics. 2024 Nov 28;12(12):862. doi: 10.3390/toxics12120862 (PMC11679399; doi:10.3390/toxics12120862)
Supplement: Supplementary file 1 [file toxics-12-00862-s001.zip › toxics-3326232-Figure S1.pdf]

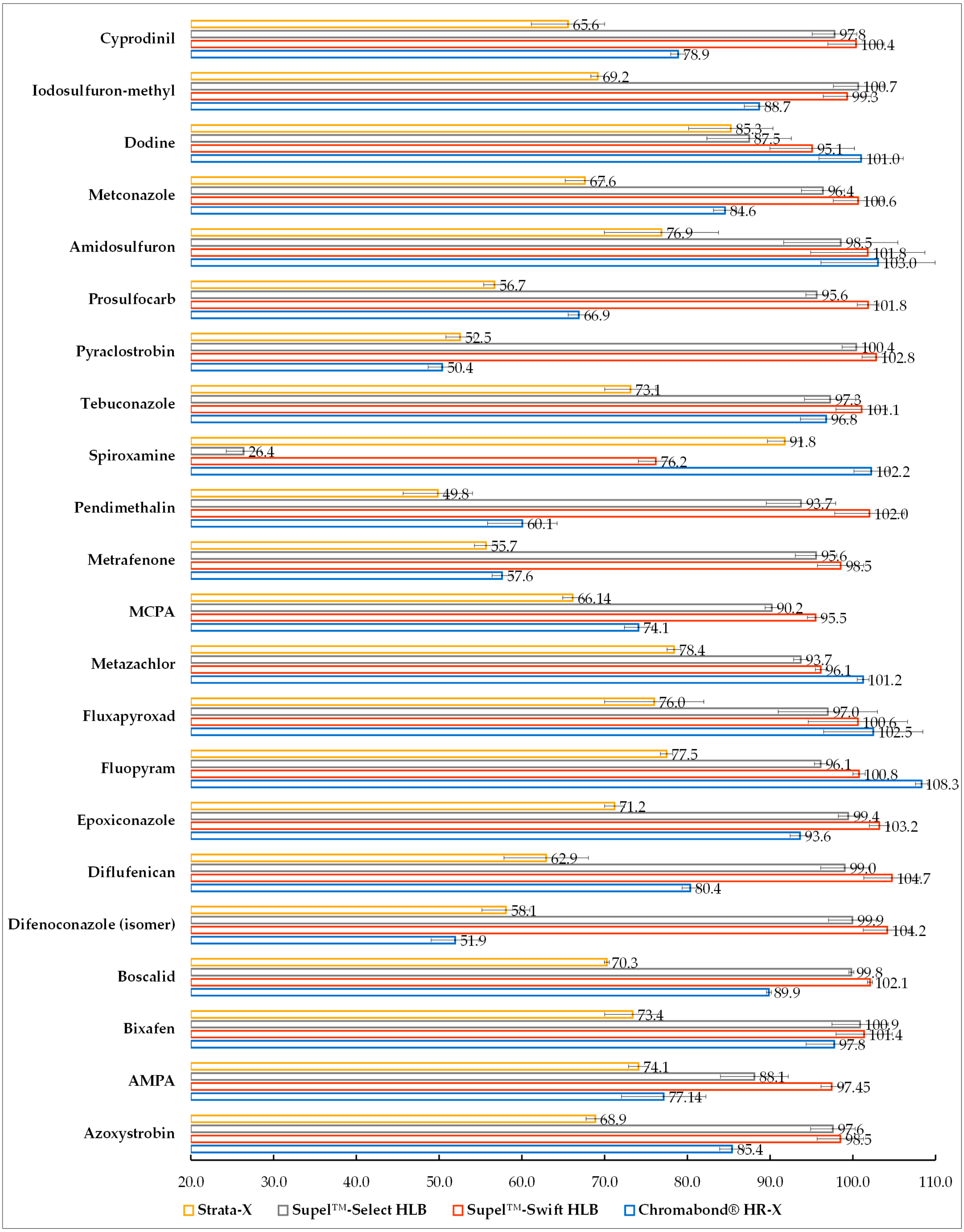

Figure S1. Recovery of pesticides from the methanolic extracts after solid-phase extraction clean-up using four SPE columns, %.
