# Supplementary material for: Pesticide Contamination of Honey-Bee-Collected Pollen in the Context of the Landscape Composition in Latvia
Source: Toxics. 2024 Nov 28;12(12):862. doi: 10.3390/toxics12120862 (PMC11679399; doi:10.3390/toxics12120862)
Supplement: Supplementary file 1 [file toxics-12-00862-s001.zip › toxics-3326232-Figure S2.pdf]

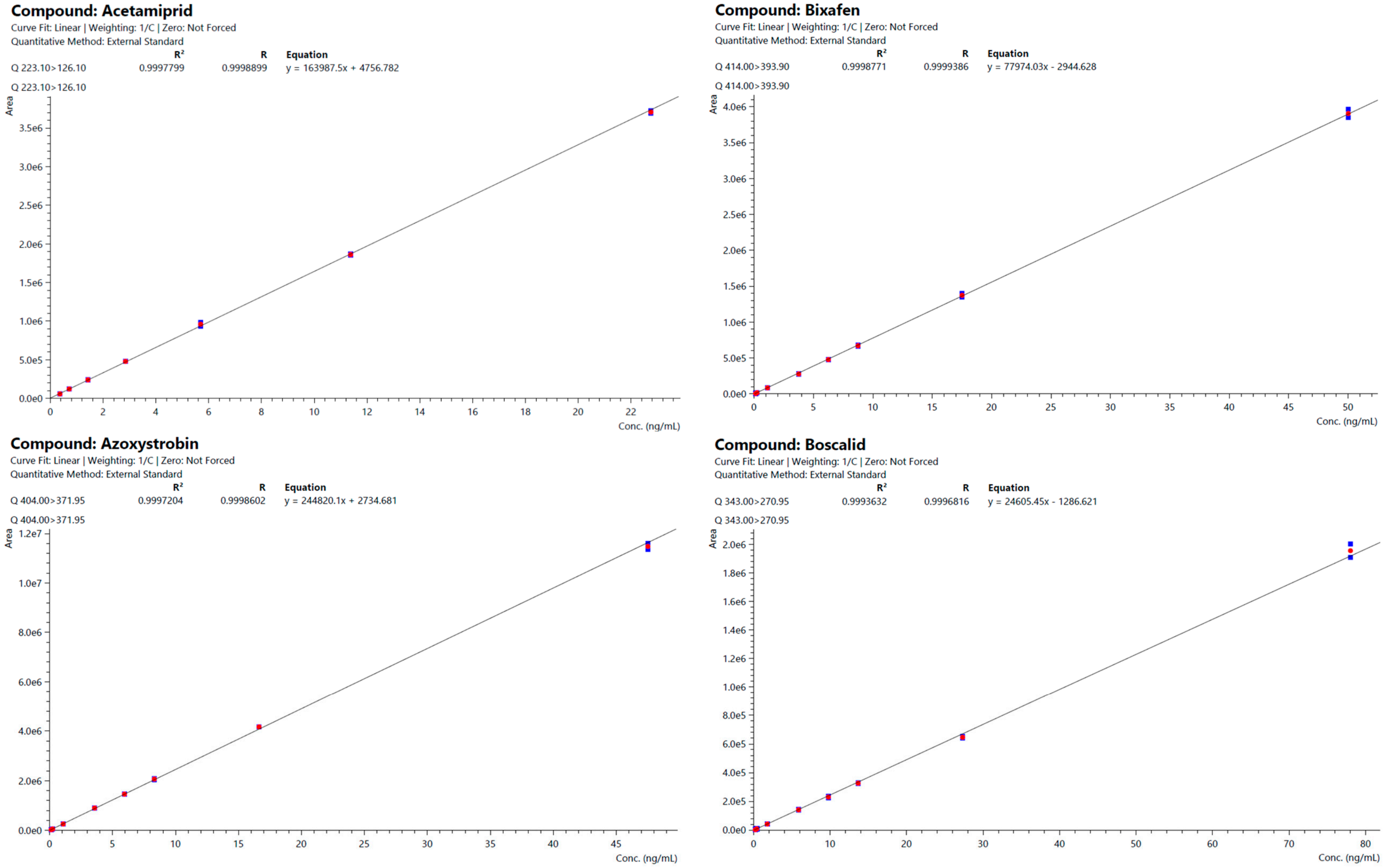

Figure S2. Calibration curves generated for major peptides detected in pollen samples. The R^2 is considered acceptable as it is higher than 0.950. The illustration was taken "LabSolutions Insight" LC-MS software version 3.7 SP3 (workstation) and generated using the quantification method.

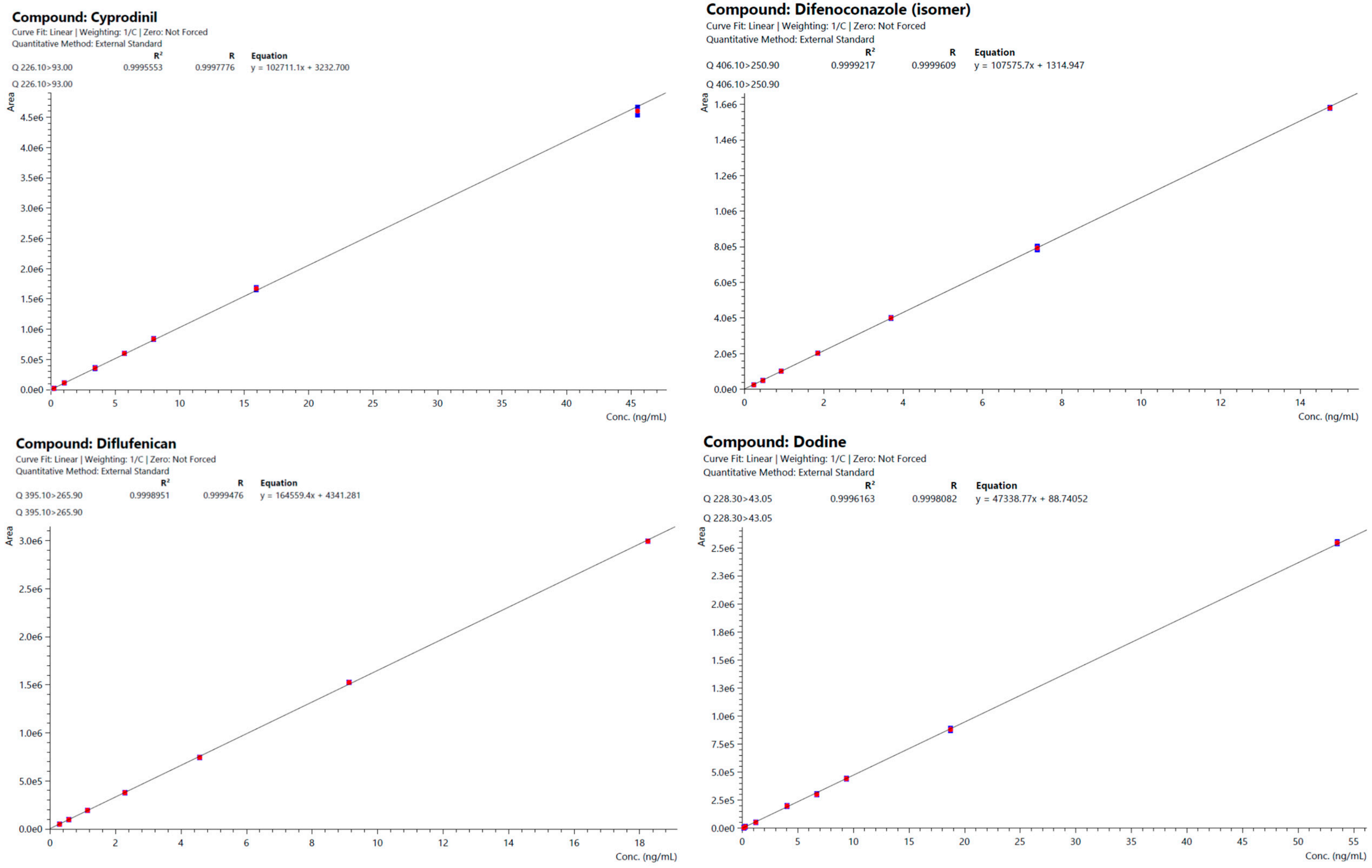

Figure S2. ***cont.*** Calibration curves generated for major peptides detected in pollen samples. The R2 is considered acceptable as it is higher than 0.950. The illustration was taken "LabSolutions Insight" LC-MS software version 3.7 SP3 (workstation) and generated using the quantification method.

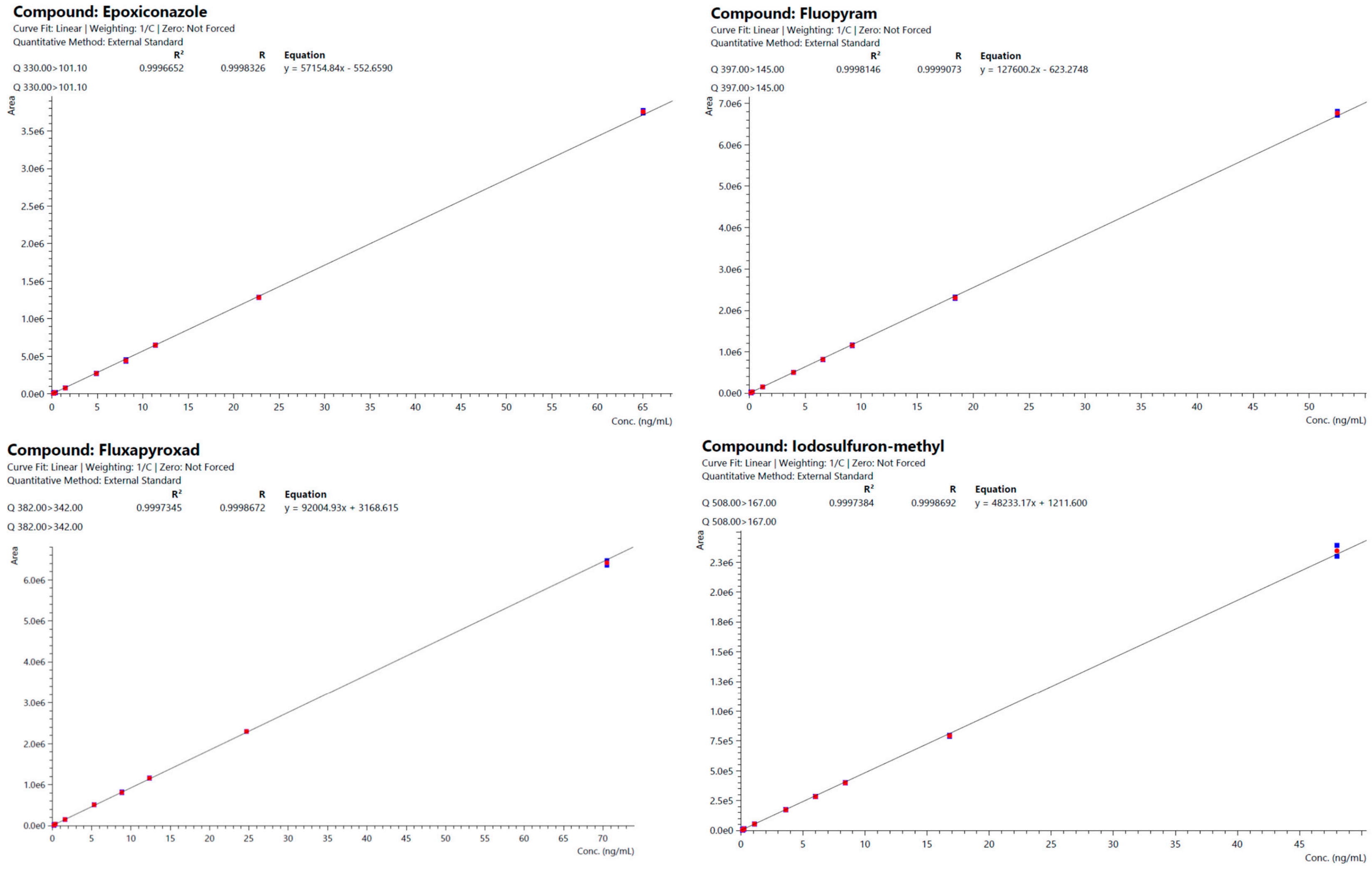

Figure S2 *cont*. Calibration curves generated for major peptides detected in pollen samples. The R^2 is considered acceptable as it is higher than 0.950. The illustration was taken "LabSolutions Insight" LC-MS software version 3.7 SP3 (workstation) and generated using the quantification method.

Compound: Metazachlor

Curve Fit: Linear | Weighting: 1/C | Zero: Not Forced
Quantitative Method: External Standard

	R^2	R	Equation
Q 277.90>210.05	0.9998205	0.9999102	y = 226144.2x + 520.5231

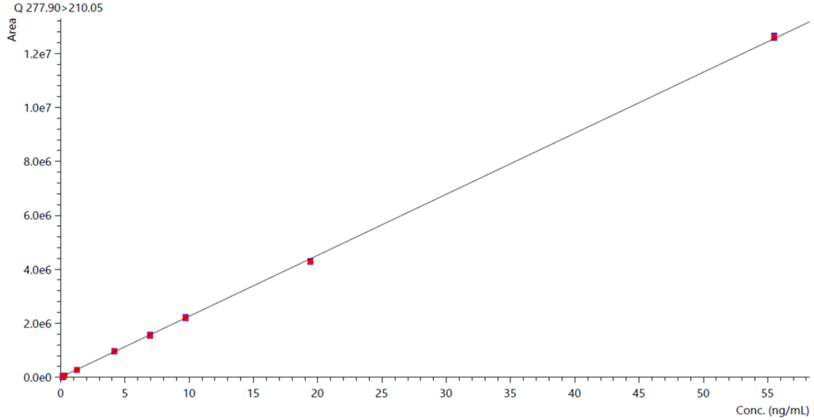

Compound: Metconazole

Curve Fit: Linear | Weighting: 1/C | Zero: Not Forced
Quantitative Method: External Standard

	R^2	R	Equation
Q 320.10>70.15	0.9997375	0.9998687	y = 59918.93x + 76.77838

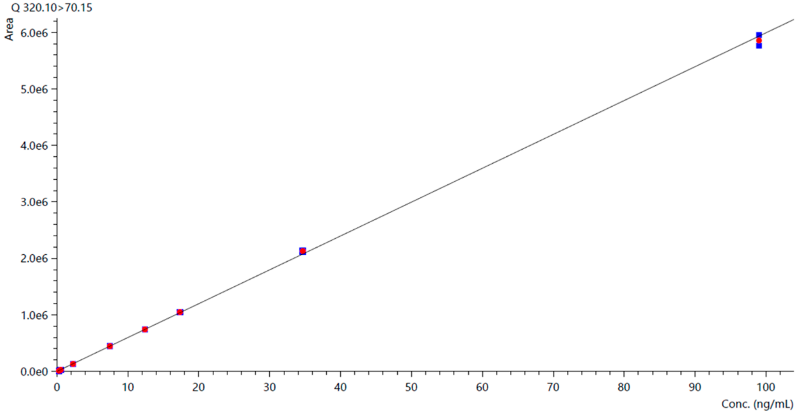

Compound: Metrafenone

Curve Fit: Linear | Weighting: 1/C | Zero: Not Forced
Quantitative Method: External Standard

	R^2	R	Equation
Q 409.00>227.00	0.9999171	0.9999586	y = 65925.61x - 1827.509

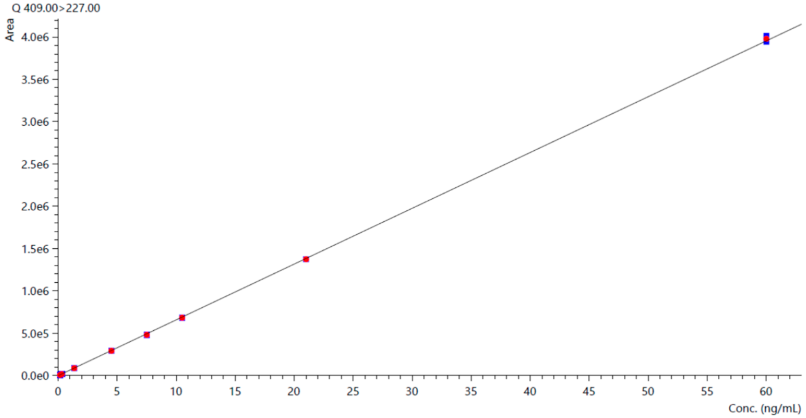

Compound: Pendimethalin

Curve Fit: Linear | Weighting: 1/C | Zero: Not Forced
Quantitative Method: External Standard

	R^2	R	Equation
Q 282.20>212.00	0.9996401	0.9998200	y = 34352.30x + 664.7764

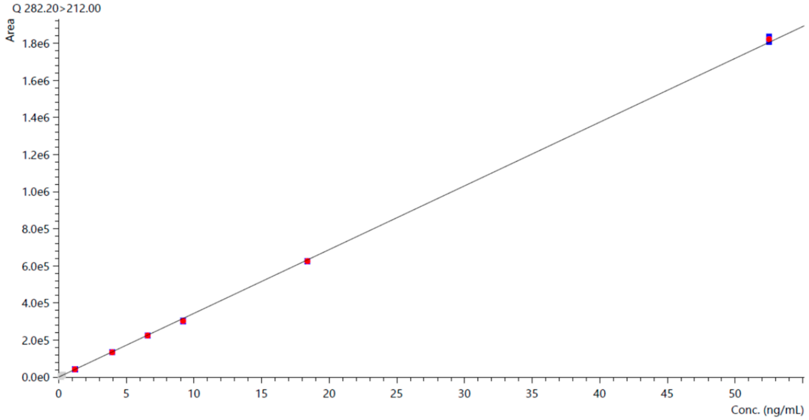

Figure S2 ***cont*****.** Calibration curves generated for major peptides detected in pollen samples. The R^2 is considered acceptable as it is higher than 0.950. The illustration was taken "LabSolutions Insight" LC-MS software version 3.7 SP3 (workstation) and generated using the quantification method.

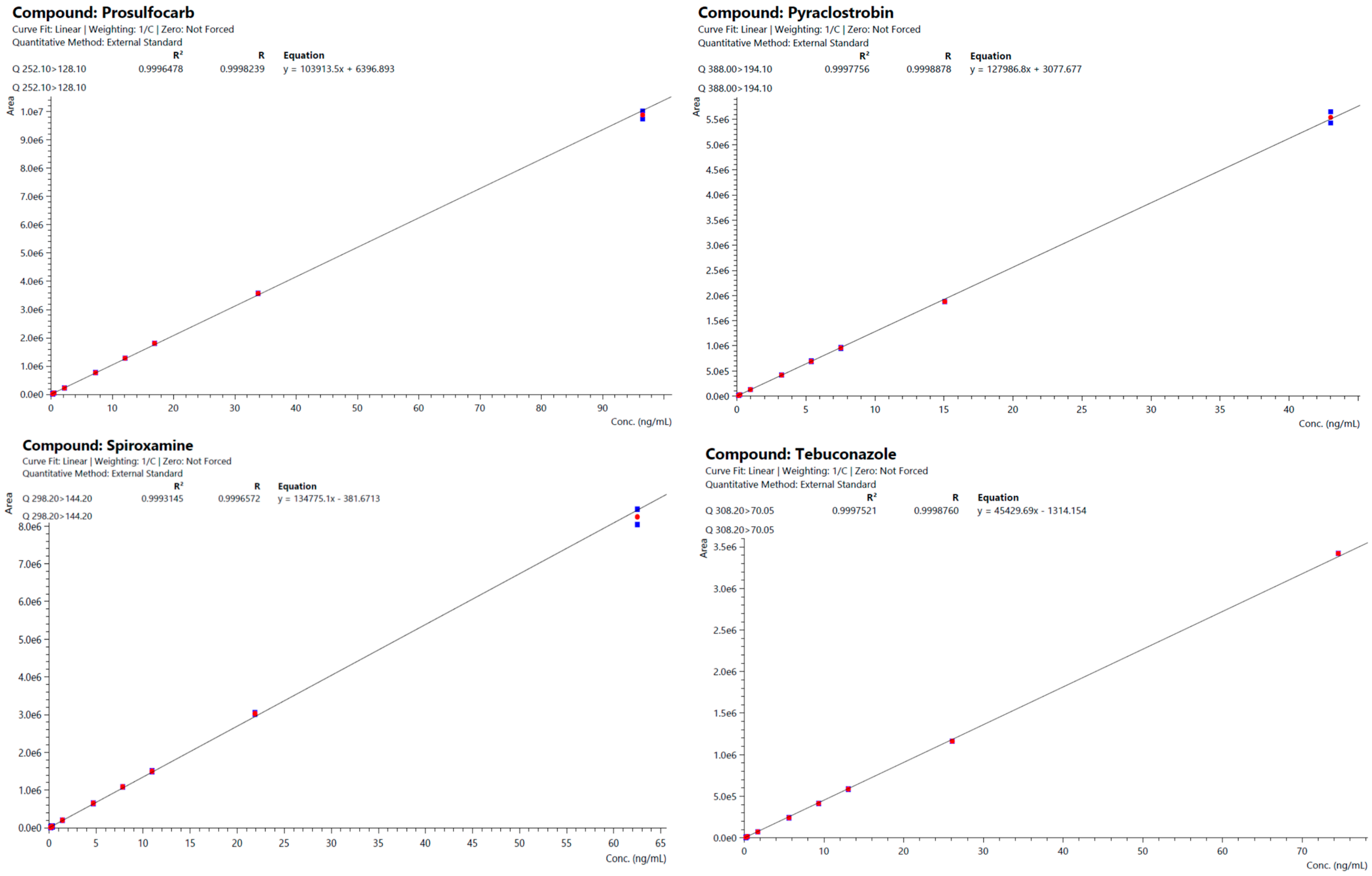

Figure S2 *cont*. Calibration curves generated for major peptides detected in pollen samples. The R^2 is considered acceptable as it is higher than 0.950. The illustration was taken "LabSolutions Insight" LC-MS software version 3.7 SP3 (workstation) and generated using the quantification method.

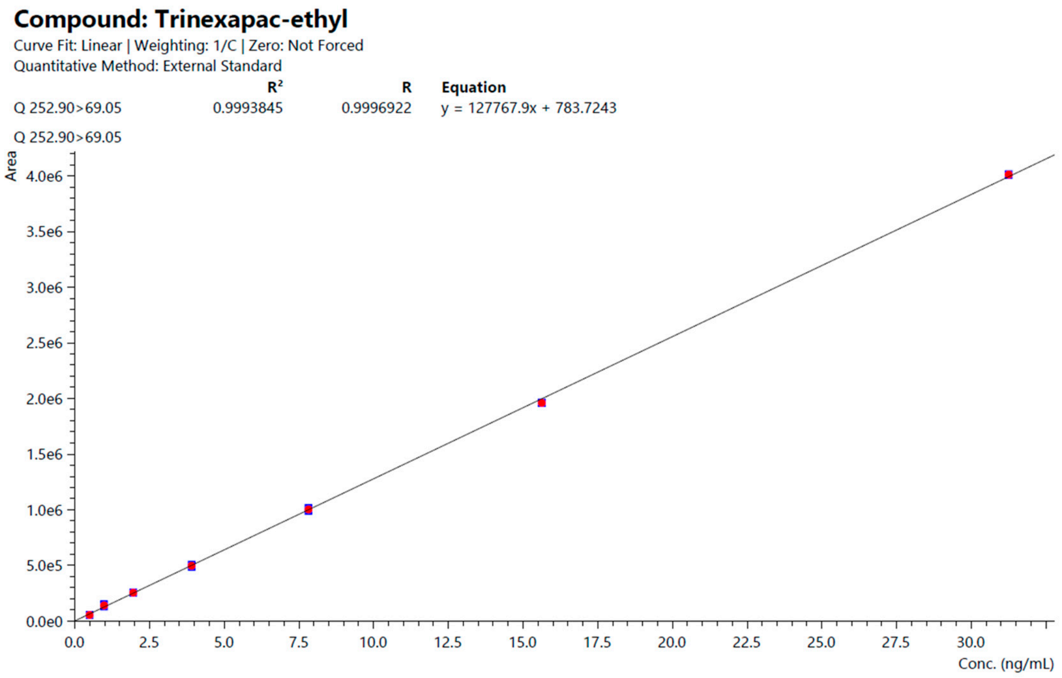

Figure S2 *cont*. Calibration curves generated for major peptides detected in pollen samples. The R^2 is considered acceptable as it is higher than 0.950. The illustration was taken "LabSolutions Insight" LC-MS software version 3.7 SP3 (workstation) and generated using the quantification method.
