# Supplementary material for: Pesticide Contamination of Honey-Bee-Collected Pollen in the Context of the Landscape Composition in Latvia
Source: Toxics. 2024 Nov 28;12(12):862. doi: 10.3390/toxics12120862 (PMC11679399; doi:10.3390/toxics12120862)
Supplement: Supplementary file 1 [file toxics-12-00862-s001.zip › toxics-3326232-Table S2.pdf]

**Table S2.** Performance of the analytical method for a particular pesticide in pollen.

| Pesticide | Pollen | | | | |
|---|---|---|---|---|---|
| | RSD (%) | LOD, mg kg⁻¹ | LOQ, mg kg⁻¹ | Linearity ($R^2$) | Mean recovery (%) |
| Acetamiprid | 2.18 | 0.000223370 | 0.000676880 | 0.9997 | 104 |
| Aminomethyl phosphonic acid (AMPA) | 5.18 | - | - | 0.9993 | 94 |
| Azoxystrobin | 2.78 | 0.000075064 | 0.000227466 | 0.9998 | 97 |
| Bixafen | 9.66 | 0.000238899 | 0.000723936 | 0.9998 | 100 |
| Boscalid | 8.54 | 0.000212800 | 0.000644848 | 0.9993 | 100 |
| Chlormequat chloride | 4.33 | - | - | 0.9991 | 77 |
| Cyprodinil | 3.11 | 0.000084564 | 0.000256253 | 0.9995 | 98 |
| Deltamethrin | 3.45 | - | - | 0.9994 | 98 |
| Difenoconazole | 14.65 | 0.000258153 | 0.000782283 | 0.9995 | 100 |
| Diflufenican | 5.14 | 0.000143075 | 0.000433562 | 0.9998 | 99 |
| Dodine | 7.00 | 0.000185936 | 0.000563442 | 0.9996 | 87 |
| Epoxiconazole | 5.51 | 0.000143795 | 0.000435742 | 0.9996 | 99 |
| Florasulam | 2.18 | - | - | 0.9997 | 97 |
| Fluopyram | 1.66 | 0.000043627 | 0.000132203 | 0.9998 | 96 |
| Fluroxypyr | 5.36 | - | - | 0.991 | 92 |
| Fluxapyroxad | 4.87 | - | - | 0.9997 | 97 |
| Iodosulfuron-methyl-sodium | 8.50 | 0.000234566 | 0.000710807 | 0.9997 | 101 |
| MCPA | 4.85 | - | - | 0.9996 | 93 |
| Metazachlor | 2.11 | 0.000055900 | 0.000169393 | 0.9998 | 94 |
| Metconazole | 2.28 | 0.000060304 | 0.000182740 | 0.9997 | 96 |
| Metrafenone | 5.62 | 0.000142668 | 0.000432326 | 0.9999 | 96 |
| Pendimethalin | 3.29 | 0.000087488 | 0.000265114 | 0.9996 | 94 |
| Prosulfocarb | 6.35 | 0.000176851 | 0.000535911 | 0.9996 | 96 |
| Pyraclostrobin | 6.22 | 0.000172821 | 0.000523701 | 0.9997 | 100 |
| Spiroxamine | 6.08 | 0.000160024 | 0.000484922 | 0.9993 | 26 |
| Tebuconazole | 3.77 | 0.000096400 | 0.000292120 | 0.9997 | 97 |
