# Supplementary material for: Pesticide Contamination of Honey-Bee-Collected Pollen in the Context of the Landscape Composition in Latvia
Source: Toxics. 2024 Nov 28;12(12):862. doi: 10.3390/toxics12120862 (PMC11679399; doi:10.3390/toxics12120862)
Supplement: Supplementary file 1 [file toxics-12-00862-s001.zip › toxics-3326232-Table S3.pdf]

Table S3. Cultivated plants in a radius of 3 km around the studied apiaries, divided by the amount of pesticide application (kilograms of active substance per hectare).

None	Low (<0.5 kg ha^{-1})	Moderate (0.5–1.0 kg ha^{-1})	High (>1.0 kg ha^{-1})
Legumes (Fabaceae)			
White melilot (*Melilotus albus*)*<br>Fodder galega (*Galega orientalis*)<br>White clover (*Trifolium repens*)*<br>Alsike clover (*Trifolium hybridum*)*<br>Red clover (*Trifolium pratense*)*<br>Fenugreek (*Trigonella foenum-graecum*)*<br>Common bean (*Phaseolus vulgaris*)*<br>Lacy phacelia (*Phacelia tanacetifolia*)*<br>Lentil (*Lens culinaris*)*<br>Alfalfa (*Medicago sativa*)<br>Lupine (*Lupinus* spp.)*<br>Birdsfoot deervetch (*Lotus corniculatus*)*<br>Vetches (*Vicia* spp.)*<br>Soybean (*Glycine max*)*<br>Common sainfoin (*Onobrychis viciifolia*)*	Pea (*Pisum sativum*)*	–	Field bean (*Vicia faba*)*
Cereals and grasses (Poaceae)			
Various grass seed crops	Oat (*Avena sativa*)<br>Spring triticale (× *Triticosecale*)<br>Mix of cereals and pulses<br>Mix of various cereals<br>Maize (*Zea mays*)*	Rye (*Secale cereale*)<br>Winter triticale (× *Triticosecale*)<br>Spring wheat (*Triticum aestivum*)<br>Spring barley (*Hordeum vulgare*)	Winter wheat (*Triticum aestivum*)<br>Winter barley (*Hordeum vulgare*)
Orchards and berry crops			
Garden rowan (*Sorbus aucuparia*)*<br>Cranberry (*Oxycoccus* spp.)*<br>Gooseberry (*Grossularia* spp.)*<br>Black chokeberry (*Aronia melanocarpa*)*<br>Guelder-rose (*Viburnum*	Raspberry (*Rubus* spp.)*<br>Strawberry (*Fragaria* × *ananassa*)*	Cherry (*Prunus* spp.)*<br>Plum (*Prunus* spp.)*	Apple (*Malus domestica*)*<br>Pear (*Pyrus communis*)*

None	Low (<0.5 kg ha^{-1})	Moderate (0.5–1.0 kg ha^{-1})	High (>1.0 kg ha^{-1})
opulus)*<br>Currants (*Ribes* spp.)*<br>Dewberry (*Rubus* spp.)*<br>Japanese quince (*Chaenomeles japonica*)*<br>Highbush blueberry (*Vaccinium corymbosum*)*<br>Grapes (*Vitis* spp.)*<br>Seabuckthorn (*Hippophae rhamnoides*)*<br>Blue honeysuckle (*Lonicera edulis*)*<br>Hazel (*Coryllus avellana*)			
Crucifers (Brassicaceae)			
Rutabaga (*Brassica napus*)<br>Turnip (*Brassica rapa*)<br>Radish (*Raphanus raphanistrum*)<br>White mustard (*Sinapis alba*)*<br>Horseradish (*Armoracia rusticana*)*	–	Cabbage (*Brassica oleracea*)	Spring rape (*Brassica napus*)*<br>Winter rape (*Brassica napus*)*
Cucurbits (Cucurbitaceae)			
Watermelon (*Citrullus lanatus*)*<br>Melon (*Cucumis melo*)*<br>Pumpkin (*Cucurbita pepo*)*<br>Cucumber (*Cucumis sativus*)*	–	–	–
Nightshades (Solanaceae)			
Eggplant (*Solanum melangena*)<br>Paprika (*Capsicum annum*)<br>Tobacco (*Nicotiana tabacum*)<br>Tomato (*Solanum lycopersicum*)	–	–	Potato (*Solanum tuberosum*)
Aster family (Asteraceae)			
Common chicory (*Cichorium intybus*)*<br>Southern globethistle (*Echinops ritro*)*<br>Tansy (*Tanacetum vulgare*)*<br>Cornflower (*Centaurea cyanus*)*<br>Common marigold (*Calendula officinalis*)*	–	–	–

**None**	**Low (<0.5 kg ha^{-1})**	**Moderate (0.5–1.0 kg ha^{-1})**	**High (>1.0 kg ha^{-1})**
Scented Mayweed (*Chamomilla recutita*)*<br>Thistle (*Silybum marianum*)*<br>Lettuce (*Lactuca sativa*)<br>Topinambur (*Helianthus tuberosus*)			
Umbellifers (Apiaceae)			
Caraway (*Carum carvi*)*<br>Dill (*Anethum graveolens*)<br>Parsnip (*Pastinaca sativa*)<br>Parsley (*Petroselinum crispum*)<br>Coriander (*Coriandrum sativum*)<br>Celery (*Apium graveolens*)	–	–	Carrot (Daucus carota)
Amaryllis family (Amaryllidaceae)			
Garlic (*Allium sativum*)<br>Leek (*Allium porrum*)	–	–	Onion (*Allium cepa*)
Various others			
Aspens (*Populus* spp.)**<br>Starflower (*Borago officinalis*)*<br>Gray alder (*Alnus incana*)**<br>Lemon balm (*Melissa officinalis*)*<br>Catnip (*Nepeta cataria*)*<br>Hemp (*Cannabis sativa*)<br>Willows (*Salix* spp.)*,**<br>Lavander (*Lavandula angustifolia*)*<br>Flax (*Linum usitatissimum*)*<br>Rhubarb (*Rheum* × *hybridum*)*<br>Sorrel (*Rumex acetosa*)<br>Asparagus (*Asparagus officinalis*)<br>Spinach (*Spinacia oleracea*)<br>Backyard gardens*,***	Buckwheat (*Fagopyrum esculentum*)*<br>Fallows*,***	–	Beetroot (*Beta vulgaris*)

* – Crops or land areas providing the honey bees with nectar and/or pollen (mentioned as 'Nectar plants' in the article).

** – Rapidly growing tree plants cultivated in the agricultural land for energetic purposes (yield is harvested every 3–5 years).

*** – Agricultural land without specific crop.
