# Supplementary material for: Pesticide Contamination of Honey-Bee-Collected Pollen in the Context of the Landscape Composition in Latvia
Source: Toxics. 2024 Nov 28;12(12):862. doi: 10.3390/toxics12120862 (PMC11679399; doi:10.3390/toxics12120862)
Supplement: Supplementary file 1 [file toxics-12-00862-s001.zip › toxics-3326232-Table S4.pdf]

Table S4. Pollen composition analysis by sampling periods, 2023.

Vecauce parish pollen origin, %	Date of sample collection						
	15.05. – 28.05.	29.05. – 11.06.	12.06. – 25.06.	26.06. – 09.07.	10.07. – 23.07.	24.07. – 06.08.	07.08. – 20.08.
Brassicaceae	40.2	78.6	27.8	31.2	20.3	2.5	–
Asteraceae	1.0	0.7	1.0	–	22.0	5.6	70.2
Fabaceae	–	–	42.0	45.1	48.4	24.8	4.0
Rosaceae	13.9	2.0	2.2	3.9	–	8.3	–
Apiaceae	–	–	12.5	10.5	–	–	–
Papaveraceae	–	–	–	–	–	34.2	–
Scrophulariaceae	–	–	–	1.3	10.6	–	–
Ranunculaceae	11.1	–	1.2	–	–	–	–
Salicaceae	25.5	5.2	–	–	–	–	–
Plantaginaceae	–	–	–	0.5	–	3.8	20.5
Other	8.3	13.5	13.3	7.5	18.5	15.0	5.0

Platone parish pollen origin, %	Date of sample collection						
	15.05. – 28.05.	29.05. – 11.06.	12.06. – 25.06.	26.06. – 09.07.	10.07. – 23.07.	24.07. – 06.08.	07.08. – 20.08.
Brassicaceae	16.2	59.7	64.9	27.9	–	–	–
Fabaceae	–	7.6	27.0	49.4	–	–	–
Rosaceae	25.4	0.4	–	2.2	–	–	–
Grossulariaceae	11.5	–	–	–	–	–	–
Lamiaceae	–	3.4	–	4.0	–	–	–
Ranunculaceae	13.3	4.0	–	–	–	–	–
Other	28.6	24.9	7.1	16.5	–	–	–

Ledurga parish pollen origin, %	Date of sample collection						
	15.05. – 28.05.	29.05. – 11.06.	12.06. – 25.06.	26.06. – 09.07.	10.07. – 23.07.	24.07. – 06.08.	07.08. – 20.08.
Brassicaceae	58.7	60.1	70.1	58.2	6.5	3.4	1.4
Asteraceae	3.6	0.2	11.9	3.9	24.5	32.8	22.6
Fabaceae	1.2	–	0.5	14.5	26.7	1.0	–
Rosaceae	19.7	–	–	10.6	7.9	–	21.2
Apiaceae	–	18.6	11.2	–	–	–	–
Primulaceae	6.9	12.5	–	–	–	–	–
Lamiaceae	1.2	–	–	5.1	11.4	16.1	10.1
Ericaceae	–	–	–	–	8.6	33.9	21.0
Hypericaceae	–	–	–	–	–	–	16.6
Other	8.7	8.6	6.3	7.7	14.4	12.8	8.0

Jelgava town pollen origin, %	Date of sample collection						
	15.05. – 28.05.	29.05. – 11.06.	12.06. – 25.06.	26.06. – 09.07.	10.07. – 23.07.	24.07. – 06.08.	07.08. – 20.08.
Brassicaceae	19.9	40.2	26.0	58.4	22.0	2.0	2.2
Asteraceae	5.9	0.5	–	1.0	3.0	42.0	72.2
Fabaceae	3.1	1.0	4.2	11.8	6.3	1.4	1.0
Rosaceae	12.4	1.3	7.0	3.4	14.6	10.6	7.6
Scrophulariaceae	3.6	22.3	14.1	–	–	–	–
Papaveraceae	1.0	–	3.4	3.2	2.2	12.2	1.2
Primulaceae	11.7	4.4	–	–	–	–	–
Ranunculaceae	14.1	4.7	17.3	–	5.0	0.4	–

Plantaginaceae	–	–	–	–	20.9	20.5	8.5
Other	28.3	25.6	28.0	22.2	26.0	10.9	7.3
**Lube parish pollen origin, %**	**Date of sample collection**						
	15.05. – 28.05.	29.05. – 11.06.	12.06. – 25.06.	26.06. – 09.07.	10.07. – 23.07.	24.07. – 06.08.	07.08. – 20.08.
Brassicaceae	65.9	73.1	62.1	5.2	1.3	0.7	23.6
Asteraceae	1.8	0.4	8.6	53.6	43.6	81.9	28.8
Fabaceae	0.5	1.2	19.5	14.8	31.2	8.0	31.3
Rosaceae	1.5	4	–	2.2	–	–	–
Apiaceae	–	–	2.0	3.8	16.4	–	–
Other	30.3	21.3	7.8	20.4	7.5	9.4	16.3
**Ambeli parish pollen origin, %**	**Date of sample collection**						
	15.05. – 28.05.	29.05. – 11.06.	12.06. – 25.06.	26.06. – 09.07.	10.07. – 23.07.	24.07. – 06.08.	07.08. – 20.08.
Brassicaceae	43.3	56.8	61.8	74.5	59.1	22.1	58.0
Asteraceae	2.9	0.2	0.9	1.4	11.0	26.8	5.8
Fabaceae	–	1.3	7.7	5.6	2.4	35.3	29.2
Rosaceae	5.6	0.5	0.9	11.6	23.1	6.4	–
Apiaceae	–	1.8	19.1	2.6	–	–	0.7
Primulaceae	27.7	21.1	2.8	–	–	–	–
Other	20.5	18.3	6.8	4.3	4.4	9.4	6.3
