# Supplementary material for: Pesticide Contamination of Honey-Bee-Collected Pollen in the Context of the Landscape Composition in Latvia
Source: Toxics. 2024 Nov 28;12(12):862. doi: 10.3390/toxics12120862 (PMC11679399; doi:10.3390/toxics12120862)
Supplement: Supplementary file 1 [file toxics-12-00862-s001.zip › toxics-3326232-Table S5.pdf]

Table S5. Calculated PHQ in pollen samples, 2023.

**Vecauce parish**	**Date of sample collection**	**15.05. – 28.05.**	**29.05. – 11.06.**	**12.06. – 25.06.**	**26.06. – 09.07.**	**10.07. – 23.07.**	**24.07. – 06.08.**	**07.08. – 20.08.**
**Active substance**	**LD50**				**PHQ**			
Acetamiprid	14.53	0.6745	2.0991	0.0551	0.0275	0.0964	0.1239	0.0895
Azoxystrobin	25.00	0.2160	1.0680	0.0760	–	–	–	–
Boscalid	166.00	0.0048	–	–	–	–	–	–
Cyprodinil	112.50	0.0027	–	–	–	–	–	–
Difenoconazole	177.00	0.0147	0.0107	–	–	–	–	–
Epoxiconazole	83.00	0.0005	–	–	–	–	–	–
Florasulam	100.00	–	–	–	–	–	–	0.0090
Fluopyram	102.30	0.0626	0.0792	–	–	–	–	–
Prothioconazole	71.00	0.0521	0.3577	0.0310	–	–	–	–
Tebuconazole	83.05	0.0072	0.0060	–	–	–	–	–
**Platone parish**	**Date of sample collection**	**15.05. – 28.05.**	**29.05. – 11.06.**	**12.06. – 25.06.**	**26.06. – 09.07.**	**10.07. – 23.07.**	**24.07. – 06.08.**	**07.08. – 20.08.**
**Active substance**	**LD50**				**PHQ**			
Acetamiprid	14.53	0.2202	0.3441	–	0.0482	–	–	–
Azoxystrobin	25.00	0.2720	0.0720	–	–	–	–	–
Bentazone	200.00	–	0.0520	–	–	–	–	–
Boscalid	166.00	–	0.0078	–	0.0030	–	–	–
Dimoxystrobin	79.40	–	0.1058	–	–	–	–	–
Fluopyram	102.30	–	0.0987	–	–	–	–	–
Prothioconazole	71.00	0.1169	0.0901	–	–	–	–	–
Spiroxamine	100.00	–	–	–	–	–	–	–
Thiacloprid	17.32	0.0982	–	0.0924	–	–	–	–
**Ledurga parish**	**Date of sample collection**	**15.05. – 28.05.**	**29.05. – 11.06.**	**12.06. – 25.06.**	**26.06. – 09.07.**	**10.07. – 23.07.**	**24.07. – 06.08.**	**07.08. – 20.08.**
**Active substance**	**LD50**				**PHQ**			
Acetamiprid	14.53	0.3923	0.6813	0.0964	0.0344	–	–	0.0413
Fluopyram	102.30	–	0.0039	–	–	–	–	–
Boscalid	166.00	0.0295	0.0096	0.0036	–	–	–	–
Picloram	63.05	–	–	–	–	–	–	0.0190
Metconazole	85.00	–	–	–	0.0082	–	–	–
Bixafen	100.00	–	0.0010	–	–	–	–	–
Pyraclostrobin	97.20	0.0267	0.0123	0.0041	–	–	–	–
**Jelgava town**	**Date of sample collection**	**15.05. – 28.05.**	**29.05. – 11.06.**	**12.06. – 25.06.**	**26.06. – 09.07.**	**10.07. – 23.07.**	**24.07. – 06.08.**	**07.08. – 20.08.**
**Active substance**	**LD50**				**PHQ**			
Acetamiprid	14.53	0.5299	0.6469	0.0826	0.8809	0.0551	0.0964	0.0482
Azoxystrobin	25.00	0.0600	0.0480	–	–	–	–	–
Boscalid	166.00	–	0.0163	0.0024	0.0241	–	–	–
Fluopyram	102.30	0.0557	0.1476	0.0401	0.0039	–	–	–
Pendimethalin	101.20	–	–	–	–	–	0.0030	0.0030
Pyraclostrobin	97.20	–	–	–	0.0216	–	–	–
Prothioconazole	71.00	0.0493	–	0.0254	–	–	–	–
Tebuconazole	83.05	0.0265	0.0096	0.0072	0.0096	–	–	–
**Lube parish**	**Date of sample collection**	**15.05. – 28.05.**	**29.05. – 11.06.**	**12.06. – 25.06.**	**26.06. – 09.07.**	**10.07. – 23.07.**	**24.07. – 06.08.**	**07.08. – 20.08.**

**Active substance**	**LD50**	**PHQ**						
Acetamiprid	14.53	2.5809	2.0165	0.1239	0.4267	0.1376	0.0344	0.1721
Boscalid	166.00	0.0910	0.0380	0.0066	0.0139	–	–	–
Dimoxystrobin	79.40	0.5542	0.2897	0.0290	0.0126	–	–	–
Metazachlor	85.00	–	–	–	–	–	–	0.0176
Pyraclostrobin	97.20	–	–	–	0.0165	–	–	–
Propikonazols	50.00	–	–	–	0.0440	–	–	–
Prothioconazole	71.00	0.0169	–	–	–	–	–	–
Spiroxamine	100.00	0.0130	0.0140	–	–	–	–	–
Tebuconazole	83.05	–	–	–	0.0012	–	–	–
**Ambeli parish**	**Date of sample collection**	**15.05. – 28.05.**	**29.05. – 11.06.**	**12.06. – 25.06.**	**26.06. – 09.07.**	**10.07. – 23.07.**	**24.07. – 06.08.**	**07.08. – 20.08.**
**Active substance**	**LD50**	**PHQ**						
Acetamiprid	14.53	0.0895	–	0.0688	0.0138	0.1721	0.0138	–
Tebuconazole	83.05	0.0289	–	–	0.0024	–	–	–
Metconazole	85.00	0.0106	–	–	–	–	–	–
Azoxystrobin	25.00	0.0040	–	–	–	–	–	–
